# Case Report: High-Calorie Glucose Infusion and Tight Glycemic Control in Ameliorating Refractory Acidosis of Empagliflozin-Induced Euglycemic Diabetic Ketoacidosis

**DOI:** 10.3389/fendo.2022.867647

**Published:** 2022-05-30

**Authors:** Siti Sanaa Wan Azman, Norlela Sukor, Muhammad Yusuf Abu Shamsi, Ilham Ismail, Nor Azmi Kamaruddin

**Affiliations:** Department of Medicine, Universiti Kebangsaan Malaysia Medical Centre (UKMMC), Kuala Lumpur, Malaysia

**Keywords:** diabetic ketoacidosis, SGLT2 inhibitor-induced diabetic ketoacidosis, euglycemic diabetic ketoacidosis, SGLT2 inhibitor, high-calorie glucose

## Abstract

The current widespread use of sodium-glucose co-transporter 2 (SGLT2) inhibitors has triggered an increase in reported cases of euglycemic diabetic ketoacidosis (EDKA), often characterized by a protracted metabolic acidosis that is resistant to conventional DKA treatment. We report a case of empagliflozin-induced EDKA with severe metabolic acidosis intractable to aggressive fluid resuscitation and boluses of bicarbonate infusion. Following the introduction of high-calorie glucose infusion coupled with tight glycemic control, the recalcitrant acidosis was successfully corrected. This is the first case report that adopts the above approach, representing a paradigm shift in the management of SGLT2 inhibitor-induced EDKA.

## Introduction

Sodium-glucose co-transporter 2 (SGLT2) inhibitors are selective inhibitors of renal glucose reabsorption and result in urinary glucose excretion of approximately 70 to 80 g/day (1). Its use in clinical practice is increasing, primarily driven by its recent established benefits in cardiovascular and renal outcome studies (2–5). SGLT2 inhibitors that are widely used include empagliflozin (10 mg and 25 mg), dapagliflozin (5 mg and 10 mg), canagliflozin (100 mg and 300 mg), and ertugliflozin (5 mg and 15 mg) daily. There have been a number of potential safety issues associated with SGLT2 inhibitors such as urinary and genital mycotic infection, risk of bone fractures, and toe amputations (6). There is also a significant increase in the reported cases of euglycemic diabetic ketoacidosis (EDKA) with an overall reported incidence of approximately 0.1% (7). Here, we report a case of severe refractory high anion gap metabolic acidosis with minimal elevation of capillary blood glucose (CBG) resulting in a delay of EDKA diagnosis. Despite aggressive hydration and repeated boluses of sodium bicarbonate infusion, the acidosis remains refractory. Only with the introduction of high-calorie glucose infusion (1 L of 10% dextrose over 8 h) together with a tighter glycemic control did the acidosis normalize within the next 4 h, thus obviating the need for dialysis.

## Case

A 60-year-old man presented to the Emergency Department with an acute onset of epigastric discomfort. His past medical history includes diabetes mellitus, hypertension, and dyslipidemia, which were diagnosed 6 years prior. Clinical examination revealed a blood pressure (BP) of 216/110 mmHg and a pulse rate of 77 bpm with the presence of a soft systolic murmur at the apex. Other systemic examinations were unremarkable. An electrocardiogram (ECG) showed ST depression at leads V4–V6 with raised troponin T of 7,121.2 pg/ml (NR: <34.2). A diagnosis of non-ST segment elevation myocardial infarction (NSTEMI) was made, and he was promptly started on double anti-platelet and anti-coagulation therapies. His other medications include metformin 500 mg twice daily, bisoprolol 5 mg daily, perindopril 8 mg daily, and amlodipine 10 mg daily. In view of his acute coronary event with a background history of diabetes, empagliflozin 25 mg daily was promptly started on the day of admission.

**Figure 1:**
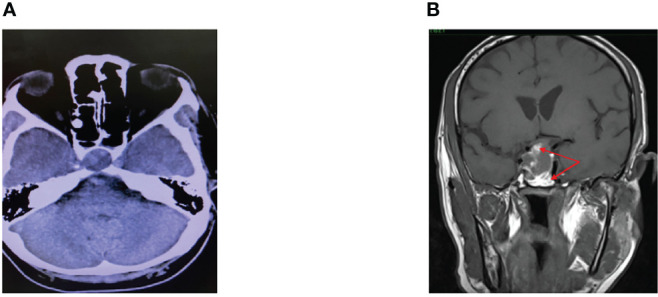
**(A)** CT scan of the brain. **(B)** MRI pituitary T1 coronal section.

Six days following admission, he complained of a sudden onset of headache associated with a complete right III, IV, and VI cranial nerve palsies. He had a spike in temperature of 38.9°C with a subsequent drop in BP. An intravenous (IV) infusion of hydrocortisone was promptly instituted, which stabilized the BP. Computed tomography (CT) scan of the brain revealed a sellar mass with an extension into the right cavernous sinus (**Figure 1A**) while the MRI of the pituitary revealed a pituitary macroadenoma, measuring 1.8 × 2.4 × 2.8 cm with extension into the suprasellar and right cavernous sinus. Within the adenoma, there were patchy hyperintensities indicating widespread bleeding in the pituitary. This was consistent with the diagnosis of pituitary apoplexy (**Figure 1B**).

His baseline cortisol level was 294 nmol/L (>50 nmol/L). The other hormonal profiles were as follows: free T4 11.37 pmol/L (9–19.05), TSH 1.35 μIU/ml (0.35–4.94), LH 4.5 IU/L, FSH 23.3 IU/L, and prolactin 0.70 μg/L (3.46–19.40).

The following morning, his general condition deteriorated with further reduction in BP necessitating inotropic support with IV noradrenaline. He was also noted to have acidotic breathing with a respiratory rate of 20 breaths/min, necessitating non-invasive ventilation (NIV). The patient’s CBG was between 8 and 14 mmol/L. There was mild acute kidney injury (AKI) with 10.7 mmol/L urea (3.2–7.4), 135 mmol/L sodium (136–145), 4.5 mmol/L potassium (3.5–5.1), and 159.3 μmol/L creatinine (63.6–110.5) together with high anion gap (26 mmol/L) metabolic acidosis with a pH of 7.059 (7.350–7.450) and a bicarbonate of 6.6 mmol/L (22.0–26.0). The lactate level was within the normal range, 0.8–1.0 mmol/L (0.5–1.6). A diagnosis of SGLT2 inhibitor-induced EDKA was confirmed by a strongly positive urinary ketone (++++). Empagliflozin was immediately withheld and insulin infusion was initiated.

His blood cultures grew Gram-positive cocci consistent with *Staphylococcus aureus* infection. The parameters of infection were elevated, with a CRP of 22.59 mg/dl (<0.5) and pro-calcitonin of 5.85 ng/ml (<0.05).

Despite aggressive fluid resuscitation with repeated colloids (gelafundin) and crystalloids (normal saline) together with multiple sodium bicarbonate infusions that spanned over 10 h, the patient remained in refractory metabolic acidosis with a pH of 7.128 and an HCO_3_ of 8.9 mmol/L (**Table 1**). At this juncture, continuous veno-venous hemofiltration (CVVH) was contemplated in view of the persistent metabolic acidosis.

**Table 1:** Serial blood investigation results and treatment timeline.

Date/Time	Day 8 of admission					Day 9 of admission		Day 10 of admission
	0930	1130	1400	1740	2200	0120	0630	0900
pH	7.059	7.059	7.128	7.128	7.279	7.338	7.323	7.393
HCO_3_ (mmol/L) (22.0–26.0)	6.6	7.4	8.0	8.9	14.1	17.5	19.0	24 **Acidosis resolved**
Blood glucose levels (mmol/L)	13	14	12	12	8.9	8.9	8.0	5.6
						Overnight range 7.7–8.5		
Ketones			Urine ++++			Urine ++		Urine negative Blood ketones 0.2 mmol/L **Ketosis resolved**
Urea (mmol/L)	11.9							9.3
Creatinine (μmol/L)	144							106.5
Lactate (mmol/L)	1.0	0.8	0.8	0.7		0.6		0.6
HCO_3_ therapy	50 ml bolus/1 h	100 ml bolus/1 h	10 ml/h maintenance infusion		–			
Treatment IV drips	Normal saline boluses with maintenance 104 ml/h		Dextrose 5% 104 ml/h	Dextrose 10% 83 ml/h				

“+” indicate presence of urine ketones. Increased number of plus signs indicate increased severity of ketosis.

At this point in time, a decision was made to initiate high-calorie glucose infusion in the form of six hourly 10% dextrose infusion (D10%). Concurrently, he was put on a tight insulin infusion with a rate of 5 units/h aiming a narrow CBG target of between 6 and 8 mmol/L. Within 4 h of initiating D10% infusion, the acidosis began to improve markedly from a pH of 7.128 to 7.279 and an HCO_3_ from 8.9 to 14.1 mmol/L (**Table 1**). With the prompt improvement of acidosis and normalization of blood ketones (0.2 mmol/L), the planned CVVH was aborted. The patient subsequently recovered and was discharged with basal bolus insulin and oral hydrocortisone (**Table 2**). A repeat MRI pituitary was planned together with a formal visual field assessment 3 months following discharge. The patient has been made aware that the introduction of empagliflozin in the setting of low-calorie intake following acute coronary syndrome, pituitary apoplexy, and subsequent sepsis has led to the development of EDKA. During his last review at 18 months follow-up, he remained well while being on insulin therapy and hydrocortisone replacement.

**Table 2:** Chronology of events from admission.

Day 1	Days 6–7	Day 8	Day 10	Days 11–22
Admission for hypertensive emergency and non-ST elevation myocardial infarction (NSTEMI). Enoxaparin, double anti-platelet, empagliflozin 25 mg daily commenced	Developed acute onset of headache and complete III cranial nerve palsy with hypotension. A diagnosis of pituitary apoplexy was made evident by clinical presentation, hormonal results, and pituitary imaging findings. Intravenous hydrocortisone initiated, patient responded, and BP stabilized.	Clinical deterioration with persistent hypotensive episodes requiring inotropic support, tachypnoeic requiring non-invasive ventilation, fever, and severe refractory metabolic acidosis with ketosis. A diagnosis of EDKA was made. Management details as illustrated in **Table 1**.	Acidosis and ketosis resolved following treatment with high-calorie glucose and insulin infusions aiming a narrow blood glucose target.	Continuation of care, which includes controlling the blood glucose with basal bolus insulin therapy, changing the intravenous to oral hydrocortisone and completion of IV antibiotics. Patient subsequently recovered and was discharged well.

## Discussion

EDKA has come into prominence with the worldwide increased use of SGLT2 inhibitors. EDKA was originally defined as diabetic ketoacidosis (DKA) with a blood glucose level of <17 mmol/L, but it is now recognized even with a blood glucose concentration of <11 mmol/L (8). Thus, the diagnosis of EDKA might be missed as a result of near-normal blood glucose levels resulting in a delay in instituting the appropriate treatment.

**Figure 2:**
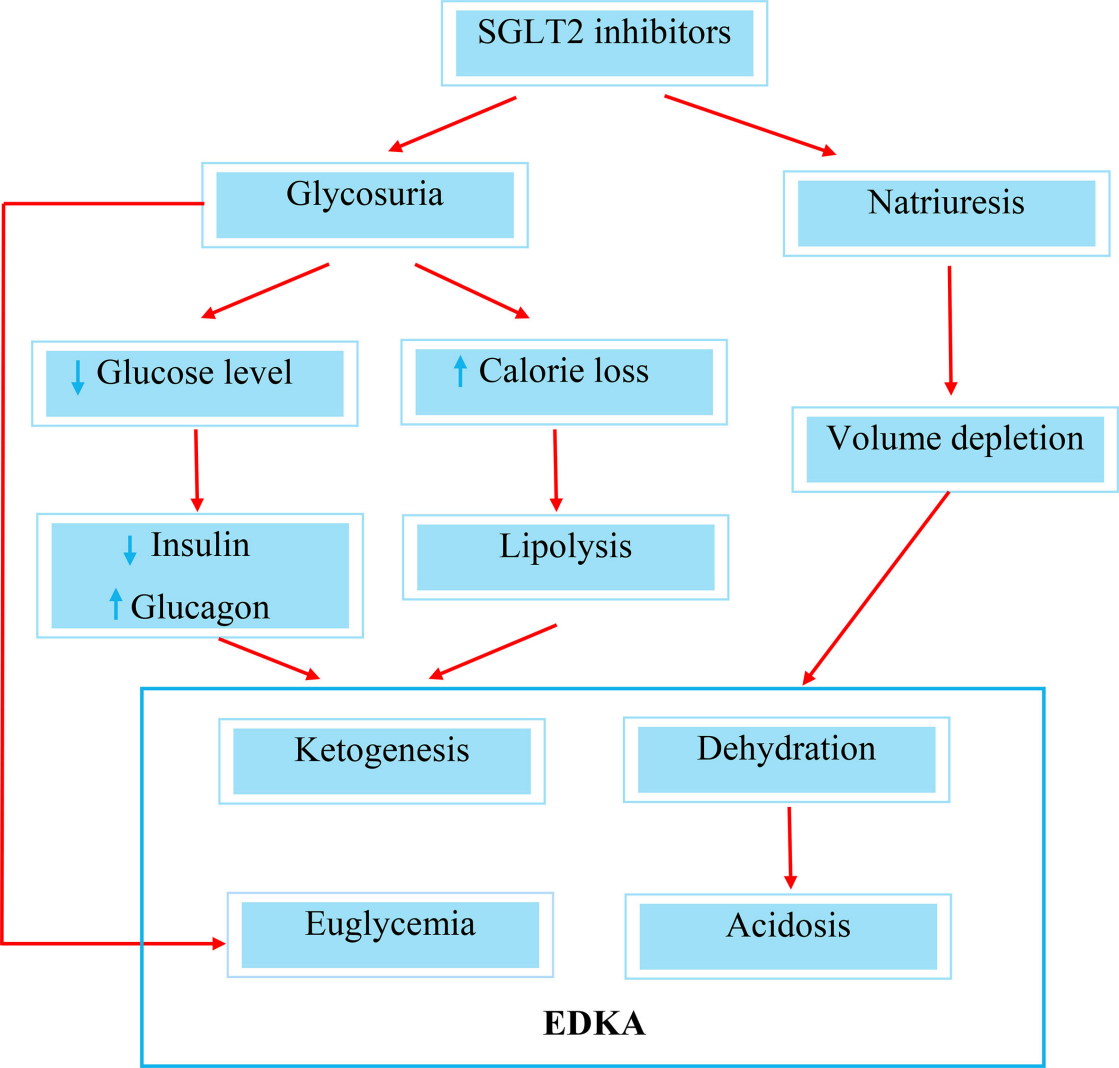
Mechanisms of SGLT2 inhibitor-induced Euglycemic Diabetic Ketoacidosis (EDKA).

SGLT2 inhibitor-induced EDKA comes about as a result of two important mechanistic pathophysiologies. Firstly, a significant calorie loss from the increased glycosuria brought about by SGLT2 inhibitors leads to lipolysis and the generation of ketone bodies. The second contributing factor is the reduction in insulin requirement following improvement in blood glucose levels (8) (**Figure 2**). In addition, the administration of SGLT2 inhibitors is associated with an increase in glucagon levels, which is mediated by the decrease in glucose levels following glucosuria. An increase in glucagon level provides a strong drive to promote the production of hepatic ketone bodies (9, 10). Ferrannini et al. conducted a study on the metabolic response to SGLT2 inhibitor in type 2 diabetic patients where they observed a reduction in insulin secretory response and an augmentation of the glucagon response, such that the prehepatic insulin-to-glucagon concentration ratio decreased by 25% with empagliflozin in response to a meal (11). Several other studies have also demonstrated a raised glucagon level with SGLT2 inhibitor administration (12, 13). This imbalance between glucagon and insulin levels is another contributing factor that leads to the development of EDKA.

Burke et al. conducted a systematic review of thirty‐four case reports of patients with type 1 and type 2 diabetes mellitus who developed EDKA while receiving SGLT2 inhibitors. Common precipitating factors identified in this systematic review included patients who had recently undergone major surgery or patients who had decreased or discontinued their insulin and patients who were diagnosed with T2DM but were subsequently found to have latent autoimmune diabetes in adulthood (14). In our patient, the likely predisposing factor was the initiation of an SGLT2 inhibitor in the setting of an acute illness (NSTEMI, septicemia, and pituitary apoplexy) where adequate calorie intake is often not the norm. An important lesson learned is that under no circumstances should the SGLT2 inhibitor be initiated in the setting of an acute illness or acute hospital admissions where adequate calorie intake is not ensured.

It is imperative to identify EDKA associated with SGLT2 inhibitors as a recent US Food and Drug Administration (FDA) review of adverse events associated with SGLT2 inhibitor use reported a fatality rate of 1.54%, as compared with 0.4% for all DKA cases (15). This adverse event was acknowledged by the regulatory bodies US FDA and European Medicines Agency (EMA), whereby they issued a safety announcement on the recognition of symptoms and related risks of this side effect. Cautions should be undertaken in conditions that restrict food intake or that can lead to severe dehydration, a sudden reduction in insulin, or an increased requirement for insulin due to illness, surgical procedure, or alcohol abuse (16, 17).

With respect to causality assessment utilizing the Naranjo adverse drug reaction probability scale (18), a score of four was obtained, which indicates a possible causal relationship. There was a temporal relationship from the time of initiation of SGLT2 inhibitors, whereby 6 days later he developed severe refractory acidosis with ketosis. Admittedly, this might have been contributed by his age, pituitary apoplexy, and sepsis. However, the severity of his acidosis could not be justified with the degree of AKI and sepsis, while his lactate level remained to be in the normal range. We were able to reverse the adverse events when empagliflozin was promptly withheld and the EDKA was managed with high-calorie glucose and insulin infusions.

Conventionally, EDKA associated with SGLT2 inhibitor use is being treated in a similar way as other types of DKA, but with consideration of the lack of hyperglycemia. Initial treatment is directed towards volume resuscitation with isotonic saline and electrolyte correction (19). The initial fluid replacement should be followed by continuous intravenous insulin infusion at a rate of 0.02–0.05 units/kg/h. Dextrose-containing fluids should be started along with the insulin infusion to avoid hypoglycemia. Serum electrolytes and glucose levels should be monitored closely during the treatment course (20).

We noticed that there were two phases in the improvement of metabolic acidosis in our patient (**Table 1**). With the initial aggressive fluid infusion and boluses of bicarbonate treatment, the pH rose from 7.059 to 7.128. Thereafter, it remained static until the high-calorie glucose infusion was introduced. We postulated that the initial improvement was solely attributed to the improvement in the hydrational status of the patient. Only with the introduction of the high-calorie glucose infusion with tight glycemic control was the other factor that led to EDKA vis-à-vis lipolysis brought under control.

We would like to highlight a report of two cases of EDKA that resulted in a prolonged intractable metabolic acidosis that lasted up to 40 h in one of the patients even though their acidosis (pH 7.27 and 7.23, respectively) was less severe than our patient (pH 7.06) (21). In one of the cases, the acidosis worsened (from pH of 7.27 to 7.1) despite being on a standard DKA treatment regimen. Similarly, we noticed that in patient 1, the blood glucose level was maintained at approximately 10 mmol/L throughout the EDKA treatment. The outcome would have been different had the high-calorie glucose infusion been instituted early in the course of the EDKA treatment together with tighter glycemic control.

Resolution of EDKA is identified by the presence of two of the following: a serum bicarbonate level ≥15 mmol/L, an anion gap ≤12 mmol/L, or a venous pH > 7.3 (20). We believe that the persistent acidosis, despite the aggressive fluid resuscitation and ongoing insulin infusion that maintained the blood glucose within the conventional guideline recommendation, was due to the intractable lipolysis that was still ongoing. This leads to inadequate calories being delivered to the tissues to switch off any lingering lipolysis. In light of the above, we have adopted two approaches, i.e., to give higher-calorie glucose infusion in order to switch off the lipolysis and to maintain tight glycemic control lower than the recommended level for critically ill patients so as to drive more glucose into the tissues and inadvertently bring to an end any persistent lipolysis and ketogenesis.

This case highlights the need to view the current EDKA management in a different light. Traditionally, the management of DKA especially in a critical illness setting requires the blood glucose to be maintained in the range of 8–10 mmol/L. Similarly, 5% dextrose infusion forms part of the integral therapy especially when the blood glucose falls below 12 mmol/L. As illustrated above, these two treatment approaches may not be adequate in addressing intractable acidosis brought about by the SGLT2 inhibitor. Only with the introduction of high-calorie glucose infusion coupled with tight glycemic control can SGLT2 inhibitor-induced lipolysis and ketogenesis be mitigated. This approach represents a paradigm shift in the management of DKA in general and SGLT2 inhibitor-induced EDKA in particular.

To date, to the best of our knowledge, this is the first case report that describes the use of high-calorie glucose infusion coupled with tight glycemic control that successfully arrests lipolysis and aborts ketogenesis in SGLT2 inhibitor-induced EDKA.

## Conclusion

High-calorie glucose infusion and tight glycemic control are key elements in ameliorating intractable metabolic acidosis brought about by SGLT2 inhibitor-induced EDKA. A randomized controlled trial or a larger case series will be needed to further consolidate this paradigm shift in the management of SGLT2 inhibitor-induced EDKA.

## Data Avalability Statement

The original contributions presented in the study are included in the article/supplementary materials, further inquiries can be directed to the corresponding author.

## Ethics Statement

Written informed consent was obtained from the individual(s) for the publication of any potentially identifiable images or data included in this article.

## Author Contributions

All authors listed have made a substantial, direct, and intellectual contribution to the work and approved it for publication.

## Conflict of Interest

The authors declare that the research was conducted in the absence of any commercial or financial relationships that could be construed as a potential conflict of interest.

## Publisher’s Note

All claims expressed in this article are solely those of the authors and do not necessarily represent those of their affiliated organizations, or those of the publisher, the editors and the reviewers. Any product that may be evaluated in this article, or claim that may be made by its manufacturer, is not guaranteed or endorsed by the publisher.
